# Higher Impact Factor of Neuroimaging Journals Is Associated With Larger Number of Articles Published and Smaller Percentage of Uncited Articles

**DOI:** 10.3389/fnhum.2018.00523

**Published:** 2019-01-04

**Authors:** Andy Wai Kan Yeung

**Affiliations:** Oral and Maxillofacial Radiology, Applied Oral Sciences, Faculty of Dentistry, The University of Hong Kong, Hong Kong, China

**Keywords:** bibliometric, citation analysis, citation distribution, impact factor, neuroimaging, uncitedness

## Abstract

The relationships among various citation metrics have been probed in multiple scientific research disciplines but not neuroimaging. The aim of the current study was to assess the citation metrics of neuroimaging journals and analyze their relationships. The Journal Citation Reports (JCRs) published by Clarivate Analytics was accessed to extract relevant data for each of the 14 journals from the neuroimaging category. Pearson correlation tests were conducted to test if the citation metrics had significant correlations. Impact factor was positively correlated with citable items (*r* = 0.717, *p* = 0.004). Percentage of uncited citable items and percentage of journal self citations were partially negatively correlated with citation distribution, i.e., the percentages of citable items that contributed to 20%, 50% and 80% of total citations. The current study has implied that all the abovementioned metrics should be considered together to provide multi-faceted evaluations instead of using a single metric, at least in the neuroimaging field.

## Introduction

Neuroimaging is one of the most exciting research fields as it probes into the human brain trying to understand the neurobiology of our behaviors and thoughts. According to 2018 Journal Citation Reports (JCRs) published by Clarivate Analytics, neuroimaging was ranked 29th out of 182 JCR journal categories in Science Citation Index Expanded (SCIE), sorted by aggregate impact factor. It had an aggregate impact factor of 3.996 based on a total of merely 14 journals, trailing behind neurosciences ranked 28th (aggregate impact factor of 4.015, 261 journals) but leading clinical neurology ranked 43th (aggregate impact factor of 3.498, 197 journals). It has been known that NeuroImage is the behemoth of the 14 neuroimaging journals that receives a dominating share of citations and is often co-cited with publications in neurosciences, clinical neurology and psychiatry (Yeung et al., 2017). For these tightly associated journal categories, it has been reported that the journal impact factor, the highly utilized and renowned metric for journal assessment, does not account for the percentage of uncited publications in a journal within a specific period of time. Larivière et al. (2009) has revealed that approximately 20% of publications in the medical field were uncited in around 2005, using a 2-year citation window. Even for Nobel laureates, 10% of their publications published in 2005 or before were still uncited at the end of 2010 (Egghe et al., 2011). Both van Leeuwen and Moed (2005) and Egghe (2010) have reported a negative correlation between impact factor and percentage of uncited publications, which is valid for the whole scientific field and numerous subcategories including neurosciences. It is advocated that a journal should be evaluated with multiple metrics concerning citation data, including those mentioned above as well as citation distribution data, such as the percentages of publications that contribute to 20%, 50% and 80% of total citations (Larivière et al., 2009). Little is known about the relationships among these bibliometric metrics for the neuroimaging journals, except that the field has been growing steadily for a decade since 2003 in terms of rising publication and citation counts and aggregate impact factor while maintaining a steady number of journals (Yeung et al., 2017). Therefore, the aim of the current study was to assess the neuroimaging journals and reveal if the relationships among the mentioned bibliometric metrics reported in other fields could be applied to neuroimaging category or not. The results should provide a more comprehensive view for readers to evaluate the performances of neuroimaging journals apart from using the single value of impact factor.

## Materials and Methods

The JCR database hosted by Clarivate Analytics was accessed on 17 July 2018. Data was extracted from JCR Year 2017 and SCIE edition. All 14 journals indexed in neuroimaging category were analyzed in terms of:

1. Impact factor 2017, inclusive of its accountable total citations (citations in 2017 to items published in 2015 and 2016) and citable items (published in 2015 and 2016);
2. Percentage of journal self citations (i.e., abovementioned total citations contributed by the journal itself);
3. Percentage of uncited citable items; and
4. Percentage of citable items that contributed to 20%, 50% and 80% of the abovementioned total citations.

Two-tailed Pearson correlation tests were conducted in SPSS 25.0 (IBM, New York, NY, USA) to test if there existed significant association among these metrics. Tests were defined as significant if *p* < 0.05.

## Results

The details of the citation metrics are listed in Table 1. The journals were listed in descending order of impact factor. As expected, the top-ranked *NeuroImage* had the largest number of total citations and citable items. Though it had the 2nd most number of uncited citable items (130), it actually had the smallest percentage of them (7.8% only). It ranked 5th in terms of percentage of journal self citations. The 2nd largest journal in terms of total citations and citable items was *American Journal of Neuroradiology*; it ranked 5th in terms of impact factor and 1st in terms of percentage of journal self citations.

**Table 1:** Various citation metrics of neuroimaging journals.

Journal	Impact factor 2017	Total citations	Citable items	Percentage of journal self citations	Percentage of uncited citable items	Percentage of citable items that contribute to		
						20%	50%	80%
						of total citations		
NeuroImage	5.426	9007	1660	0.153	0.078	0.047	0.211	0.536
Human Brain Mapping	4.927	1363	325	0.164	0.157	0.040	0.135	0.265
Neuroimage Clinical	3.869	1803	466	0.050	0.122	0.049	0.206	0.485
Brain Imaging and Behavior	3.719	740	199	0.047	0.211	0.070	0.291	1.000
American Journal of Neuroradiology	3.653	2645	724	0.096	0.182	0.040	0.181	0.482
Journal of Neurointerventional Surgery	3.524	1702	483	0.206	0.269	0.050	0.201	0.681
Journal of Neuroradiology	2.706	230	85	0.339	0.247	0.071	0.271	1.000
Psychiatry Research: Neuroimaging	2.455	793	323	0.053	0.204	0.043	0.186	0.449
Neuroradiology	2.346	596	254	0.062	0.260	0.039	0.177	0.457
Journal of Neuroimaging	1.952	504	258	0.026	0.302	0.043	0.171	0.419
Clinical EEG and Neuroscience	1.807	159	88	0.025	0.420	0.023	0.136	0.511
Stereotactic and Functional Neurosurgery	1.648	178	108	0.045	0.352	0.037	0.148	0.380
Neuroimaging Clinics of North America	1.275	102	80	0.010	0.400	0.050	0.163	0.363
Klinische Neurophysiologie	0.158	9	57	0.333	0.912	0.018	0.070	1.000

Please note that the summation of citations received by all citable items is less than the number of total citations, as the latter include citations to non-“citable items.”

Pearson correlation tests have revealed that impact factor was positively correlated with the number of citable items (*r* = 0.717, *p* = 0.004), and negatively correlated with percentage of uncited citable items (*r* = −0.827, *p* < 0.001). A scatter plot of log-10 impact factor against percentage of uncited citable items (Supplementary Figure S1) was prepared in a similar fashion as van Leeuwen and Moed (2005; see their Figures 1, 2). If a neuroimaging journal had an impact factor approaching 1, its percentage of uncited citable items would be in the range of 40% to 50%. If it had an impact factor approaching 0.1, the percentage would be greater than 90%.

Meanwhile, percentage of uncited citable items was partially negatively correlated with citation distribution, namely with percentage of citable items that contributed to 20% and 50% of total citations (*r* = −0.581, *p* = 0.029 and *r* = −0.658, *p* = 0.010 respectively; Table 2).

**Table 2:** Pearson correlation coefficients between various citation parameters of neuroimaging journals.

			Impact factor 2017	Citable items	Percentage of journal self citations	Percentage of uncited citable items	Percentage of citable items that contributed to		
							20%	50%	80%
							of total citations		
Impact factor 2017			/						
Citable items			0.717**						
Percentage of journal self citations			−0.012	0.028					
Percentage of uncited citable items			−0.827**	−0.515	0.364				
Percentage of	20%		0.435	0.083	0.048	−0.581*			
citable items	50%	of total citations	0.503	0.21	−0.043	−0.658*	0.934**		
that contributed to	80%		−0.168	−0.166	0.642*	0.384	0.329	0.363	/

**p < 0.01, *p < 0.05.

## Discussion

The current study has revealed the performance of neuroimaging journals in terms of citation metrics. Though there is limited data in the existing literature confirming or rebutting the relationship between impact factor and the number of published articles (citable items), the current results have implied that, at least for neuroimaging journals, size really matters.

Based on the data reported by van Leeuwen and Moed (2005) and Egghe (2010) has established that the impact factor of journals and their uncitedness factor (i.e., the percentage of uncited articles) follows a relationship best described with a horizontal S-shape curve. At a first glance, the Supplementary Figure S1 of the current study seemed not to demonstrate the characteristic S-shape. Upon a closer examination of the original data presented by van Leeuwen and Moed (2005), the neuroimaging journal data from the current study actually conformed to the middle segment of the S-shape, which was a relatively straight line when the impact factor range was between 0.1 and 10 (see their Figures 1, 2). This straight segment held true for the overall SCI publications and subcategories namely the biochemistry and molecular biology, inorganic chemistry, physical chemistry (van Leeuwen and Moed, 2005), as well as immunology and surgical journals (Weale et al., 2004). Our interpolations that impact factor approaching 1 and 0.1 would imply percentage of uncited citable items being 40%–50% and >90% were also consistent to their findings (van Leeuwen and Moed, 2005). Without the existence of journals with extremely large (>10) or small (<0.1) impact factor, the plot representing neuroimaging journals did not have the curved segments at the extremities and thus the Pearson correlation coefficient between these two metrics, −0.827, was larger than that of neurosciences (−0.64), general and internal medicine (−0.69) and the overall SCI category (−0.63) reported by them (van Leeuwen and Moed, 2005).

Next, the magnitude of uncitedness was compared to existing studies. In the dated literature, it was reported that there existed 0.45%–49.9% of articles that remained uncited at least for 4 years after publication in the biomedical fields ranging from chemistry to pharmacology (Stern, 1990). A recent report has revealed that the percentages of articles published in 2000 remained uncited 1 year and 2 years after publication were around 40%–80% and 20%–60% respectively, with biology articles having percentages of 40% and ~20% respectively (van Noorden, 2017). These studies have shown that the proportions of uncited articles in various research fields were largely diversified. The survey by Larivière et al. (2009) has found that the medical field publications had 20% of uncitedness in 2005 using a 2-year citation window, which has been decreasing over time. Weale et al. (2004) similarly concluded that 23.7% of articles published in immunology and surgical journals were uncited. Within the 14 journals of the neuroimaging field, the current results reported the uncitedness ranging from 7.8% to 91.2%, and an overall of 18.2%. This was somehow comparable to the findings by Larivière et al. (2009).

Meanwhile, in the medical field the percentages of articles that contributed to 20%, 50% and 80% of total citations were 2%, 11% and 33% respectively (Larivière et al., 2009). For immunology and surgical journals, the percentages of articles that contributed to 50% of total citations were 15%–18% (Weale et al., 2004). For a cardiovascular journal, 14% of articles contributed to 50% of the total citations (Opthof et al., 2004). If the percentages from each neuroimaging journal were averaged, the respective equivalent values would be 4.4%, 18.2% and 57.3%. This implied that citations to publications from neuroimaging journals were probably more dispersed than those in the medical field. From Table 2, one would realize that impact factor had no significant correlation with any of these three percentages, implying that these metrics might indeed reveal unique assessment features distinctive from the impact factor. These three percentages did have partial correlation with the percentage of journal self citations and uncited citable items (Table 2). However, the exact relationships remained to be established. One important consideration for this, and also limitation of the current analysis, is that the citation count used to calculate impact factor includes citations to publications that are not counted as citable items (Moed and Van Leeuwen, 1995, 1996). Therefore, a journal editor could boost its impact factor by publishing non-“citable items” that self cite a lot of its “citable items” (Falagas and Alexiou, 2008), which complicates the underlying behavior of citations. A study by Opthof revealed that such journal self citation practice in cardiovascular science could inflate the journal impact factor by as much as 69% (Opthof, 2013). Another limitation is the small sample size of *n* = 14. This study is similar to previous studies in evaluating the relationship of journal impact factor and other metrics by considering a particular JCR journal category. Because the Neuroimaging category consists of 14 journals only, the current sample size already covers the entire category. It is a common practice to use impact factor to evaluate journals, validate scientific relevance of researchers or research programs and therefore decide employment, funding and tenure (Hecht et al., 1998). Meanwhile, the San Francisco Declaration on Research Assessment (DORA^1^) developed in 2012 urged for the considerations of scientific content of articles rather than publication metrics for hiring, tenure and promotion decisions. Though the current study was not aimed to judge the appropriateness of such usage of the impact factor, the current results have implied that all the abovementioned journal metrics should be considered together, instead of a single metric, to provide a more comprehensive understanding of journal performance, at least in the neuroimaging field. Their extended use for evaluation purposes of individual researchers, however, may be deemed too advanced and complex. For instance, uncited articles may be “sleeping beauties,” which can be uncited for a long time, but eventually highly cited (van Raan, 2004; Yeung and Ho, 2018). It is unfeasible to evaluate researchers based on complex metrics that may not truly reflect the scientific value of their work.

## Conclusions

The current study has evaluated the relationships among various citation metrics for the neuroimaging journals. Impact factor positively correlated with the number of published citable items, but negatively correlated with percentage of uncited citable items. Percentages of citable items that contributed to 20%, 50% and 80% of total citations did not correlate with impact factor. These metrics reflecting citation distribution should be considered together with other citation metrics when one is assessing neuroimaging journal performance.

## Data Availability

All datasets generated for this study are included in the manuscript and the supplementary files.

## Author Contributions

The author confirms being the sole contributor of this work and has approved it for publication.

## Conflict of Interest Statement

The author declares that the research was conducted in the absence of any commercial or financial relationships that could be construed as a potential conflict of interest.

## Footnotes

^1^https://sfdora.org/read/

## Supplementary Material

FIGURE S1 Scatter plot of log-10 impact factor 2017 against percentage of uncited citable items.
