# Supplementary figures and images for: Higher Impact Factor of Neuroimaging Journals Is Associated With Larger Number of Articles Published and Smaller Percentage of Uncited Articles
Source: Front Hum Neurosci. 2019 Jan 4;12:523. doi: 10.3389/fnhum.2018.00523 (PMC6338050; doi:10.3389/fnhum.2018.00523)

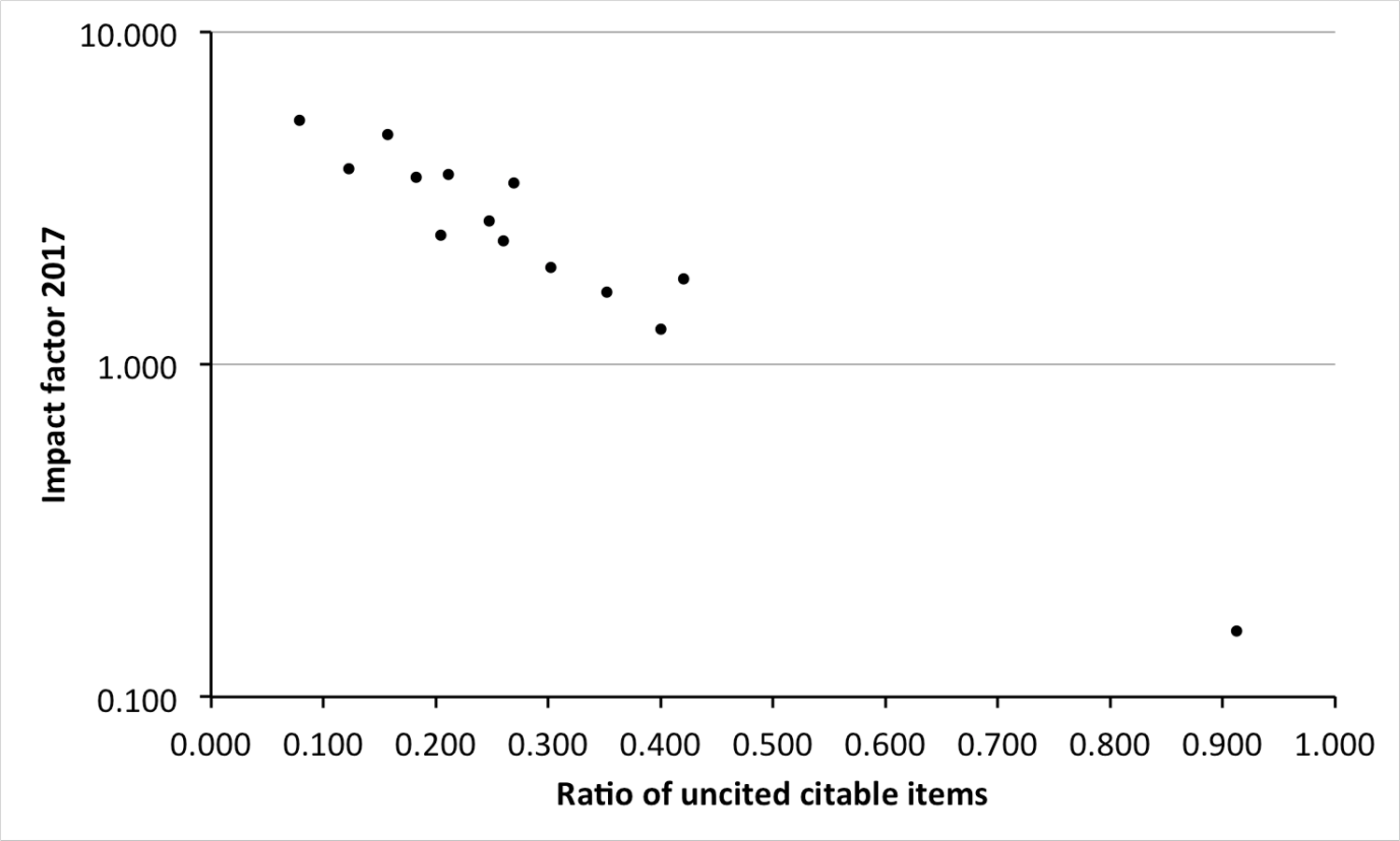

Supplement: FIGURE S1 — Scatter plot of log-10 impact factor 2017 against percentage of uncited citable items. [file Image_1.TIFF]
